# Supplementary material for: Patterns of Intron Gain and Loss in Fungi
Source: PLoS Biol. 2004 Nov 30;2(12):e422. doi: 10.1371/journal.pbio.0020422 (PMC532390; doi:10.1371/journal.pbio.0020422)
Supplement: Table S1 — Also available at http://genes.mit.edu/NielsenEtAl/. (4.3 MB ZIP). [file pbio.0020422.st001.zip › NielsenEtAl/html/287.html]

AN5282.1.NCU09680.1.MG05520.1.FG03628.1


```
 CLUSTAL W (1.82) Multiple Sequence Alignments - Introns Inserted


Sequence 1: NCU09680.1	484 aa
Sequence 2: FG03628.1	458 aa
Sequence 3: MG05520.1	487 aa
Sequence 4: AN5282.1	450 aa
Alignment Length: 494 aa
Number Identitical Residues: 202 aa
Alignment Score (without introns) 10552


MG05520.1 	-MASKLFLAAALLQGALSSPLAVEERQACA-AQW2GQC~GGQDYTGPTCCQSGSTCVVSN
NCU09680.1	MAAKKLLLAAALTASALAAP-VLEDRQNCG-SAW2SQC~GGIGWSGATCCSSGNSCVEIN
FG03628.1 	MTAYKLFLAAAFAATALAAP-VEE-RQSCSNGVW2SQC~GGQNWSGTPCCTSGNKCVKVN
AN5282.1  	MHYSASGLALAFLLPAIQAQ----------QTLY~GQC1GGSGWTGATSCVAGAACSTLN
          	       ** *:   *: :           .  : .** ** .::*...* :*  *   *

MG05520.1 	QW~YSQCLPGSSNPTTTSRTSTSSSSSTSRTSSSTSRPPSSVPTTPTSVPPTITTTPTTT
NCU09680.1	SY~YSQCLPGA-QVTTTAGASSTSPTSTSKVSSTTSK----VTSSSAAQPITTTTAPSVP
FG03628.1 	DF~YSQCQP---------GSADPSPTST-IVSATTTK----ATTTGSGG--SVTSPPPVA
AN5282.1  	QW1YAQCLP---------AATTTSTTLTTTTSS--------VTTTSNPGSTTTTSSVTVT
          	.: *:** *          :: .*.: *: .*:        ..::    . : *:. ...

MG05520.1 	PTG-GSGPGTTASFTGNPFAGVNLFPNKFYSSEVHTLAIPSLTGSLVAKASAVAQVPS~F
NCU09680.1	TTTIAGGASSTASFTGNPFLGVQGWANSYYSSEIYNHAIPSMTGSLAAQASAVAKVPT~F
FG03628.1 	TN--------------NPFSGVDLWANNYYRSEVSTLAIPKLSGAMATAAAKVADVPS~F
AN5282.1  	ATA-----------SGNPFSGYQLYVNPYYSSEVQSIAIPSLTGTLSSLAPAATAAAK0T
          	..            :.*** * : : * :* **: . ***.::*:: : *. .: ...  

MG05520.1 	QWL~DIAAK0VETLMPGALADVRAANAAGGN--YAAQLV2VYDLPDRDCAAAASNGEFSI
NCU09680.1	QWL~DRNVT~VDTLMKSTLEEIRAANKAGANPPYAAHFV~VYDLPDRDCAAAASNGEFSI
FG03628.1 	QWM2DTYDH~IS-FMEESLADIRKANKAGGN--YAGQFV~VYDLPDRDCAAAASNGEYSL
AN5282.1  	R--~DVAAK~VP-TMATYLADIRSQNAAGANPPIAGQFV~VYDLPDRDCAALASNGEFAI
          	:   *     :   *   * ::*  * **.*.. *.::* *********** *****:::

MG05520.1 	ADGGVVKYKAYIDAIRKQLLAYSDVRTILVI0E~PD1SLANMVTNMGVPKC1AGAKDAYL
NCU09680.1	ANGGVANYKTYINAIRKLLIEYSDIRTILVI~E~PD~SLANLVTNTNVAKC~ANAASAYR
FG03628.1 	DKDGKNKYKAYIAKIKGILQDYSDTRIILVI~E1PD~SLANMVTNMNVPKC~ANAASAYK
AN5282.1  	SDGGVQHYKDYIDSIREILVEYSDVHVILVI~E1PD~SLANLVTNLNVAKC~ANAQSAYL
          	 ..*  :** **  *:  *  *** : **** * ** ****:*** .*.** *.* .** 

MG05520.1 	ECTIYAVKQLNLPHVAMYLDGG~HAGWLGWPANLQPAADLFGKLYADAGKPSQLRGMA0T
NCU09680.1	ECTNYAITQLDLPHVAQYLDAG~HGGWLGWPANIQPAATLFADIYKAAGKPKSVRGLV~T
FG03628.1 	ELTIHALKELNLPNVSMYIDAG~HGGWLGWPANLPPAAQLYGQLYKDAGKPSRLRGLV~T
AN5282.1  	ECTNYAVTQLNLPNVAMYLDAG1HAGWLGWPANLQPAANLYAGVYSDAGSPAALRGLA~T
          	* * :*:.:*:**:*: *:*.* *.********: *** *:. :*  **.*  :**:. *

MG05520.1 	NVANYNAWDLTTAPSYT0TPNPNFDEKKYISAFAPLLAAKGWS-AHFIIDQG1RSGKQPT
NCU09680.1	NVSNYNGWSLSSAPSYT~TPNPNYDEKKYIEAFSPLLNAAGFP-AQFIVDTG~RSGKQPT
FG03628.1 	NVSNYNAWKLSSKPDYT~ESNPNYDEQKYIHALSPLLEQEGWPGAKFIVDQG~RSGKQPT
AN5282.1  	NVANYNAWAIDTCPSYT~QGNSVCDEKDYINALAPLLRAQGFD-AHFITDTG1RNGKQPT
          	**:***.* : : *.**   *.  **:.** *::***   *:  *:** * * *.*****

MG05520.1 	GQKEWGHWCNQQGVGFGRRPSANTGSELADAFVWIKPGGECDGVSDPTAPRFDHFCGTDY
NCU09680.1	GQIEQGDWCNAIGTGFGVRPTTNTGSSLADAFVWVKPGGESDGTSDTSATRYDYHCGLS-
FG03628.1 	GQKAWGDWCNAPGTGFGLRPSANTGDALVDAFVWVKPGGESDGTSDTSAARYDYHCGID-
AN5282.1  	GQQAWGDWCNVIGTGFGARPSTNTGDSLLDAFVWVKPGGESDGTSDTSAARYDAHCGYS-
          	**   *.***  *.*** **::***. * *****:*****.**.**.:*.*:* .** . 

MG05520.1 	GAMS~DAPQAGQWFQ~KYFEMLLTNANPPL-
NCU09680.1	DALK~PAPEAGQWFQ~AYFEQLLKNANPAF-
FG03628.1 	GAVK2PAPEAGTWFQ~AYFEQLLKNANPSFL
AN5282.1  	DALQ~PAPEAGTWFQ0AYFVQLLQNANPSF-
          	.*:.  **:** ***  **  ** ****.:
```
